# ENSO-driven climate variability promotes periodic major outbreaks of dengue in Venezuela

**DOI:** 10.1038/s41598-018-24003-z

**Published:** 2018-04-10

**Authors:** M. F. Vincenti-Gonzalez, A. Tami, E. F. Lizarazo, M. E. Grillet

**Affiliations:** 1 Department of Medical Microbiology, University of Groningen, University Medical Center Groningen, Groningen, The Netherlands; 2 Laboratorio de Biología de Vectores y Parásitos, Instituto de Zoología y Ecología Tropical, Facultad de Ciencias, Universidad Central de Venezuela, Caracas, Venezuela; 3 Departamento de Parasitología, Facultad de Ciencias de la Salud, Universidad de Carabobo, Valencia, Venezuela

## Abstract

Dengue is a mosquito-borne viral disease of global impact. In Venezuela, dengue has emerged as one of the most important public health problems of urban areas with frequent epidemics since 2001. The long-term pattern of this disease has involved not only a general upward trend in cases but also a dramatic increase in the size and frequency of epidemic outbreaks. By assuming that climate variability has a relevant influence on these changes in time, we quantified the periodicity of dengue incidence in time-series of data from two northern regions of Venezuela. Disease cycles of 1 and 3–4 years (*p* < 0.05) were detected. We determined that dengue cycles corresponded with local climate and the El Niño Southern Oscillation (ENSO) variation at both seasonal and inter-annual scales (every 2–3 years). Dengue incidence peaks were more prevalent during the warmer and dryer years of El Niño confirming that ENSO is a regional climatic driver of such long-term periodicity through local changes in temperature and rainfall. Our findings support the evidence of the effect of climate on dengue dynamics and advocate the incorporation of climate information in the surveillance and prediction of this arboviral disease in Venezuela.

## Introduction

Dengue is one of the fastest spreading mosquito-borne diseases worldwide. Annually, 390 million people become infected with one of four serologically distinct serotypes of dengue virus (DENV-1 to -4), through the bites of infected females of *Aedes aegypti*^1,2^. This neglected tropical disease (NTD) has shown a 30-fold increase in global incidence over the past 50 years, including its severe form, dengue haemorrhagic fever^3^ and prospective studies have already predicted a dramatic increase of dengue in the following 30 years^4^. The constant increase of dengue risk and dengue epidemics has been the target of a number of studies^5,6^ but dengue transmission dynamics can be highly heterogeneous due to the complex interactions among virus serotypes, vector, and host.

Previous studies indicate that dengue may have seasonal and inter-annual patterns of occurrence^7–10^. At a seasonal scale, disease incidence is characterized by annual regular cycles. The strongest external drivers of this variation are the corresponding annual changes in rainfall and temperature, the main factors that affect the ecology of *Aedes* mosquitoes and the virus. Both variables influence mosquito reproduction and mortality rates, the blood feeding frequency of the *Aedes* female and the extrinsic incubation period of the virus which in turn, determines the degree and shape of human exposure to that infection^11,12^. At an inter-annual scale, major disease cycles have been observed every few years in some epidemiological settings, being those periodic epidemics more complex to account for^8,13,14^. Some studies have associated these periodic outbreaks with global inter-annual climatic variations such as El Niño Southern Oscillation (ENSO)^14–16^, since ENSO determines periodic changes in local climatic variables. Intrinsic factors like host immunity and host susceptibility have also been identified as explanatory factors of these periodic epidemics^17^. Finally, a more comprehensive approach appeals to combine both extrinsic and intrinsic mechanisms to account for these inter-annual epidemic cycles^18^.

Dengue is one of the NTDs of major public health importance in Venezuela, showing an increment in incidence, magnitude and frequency of outbreaks during the last years. The four DENV serotypes circulate in the country. Additionally, between 2014 and 2016, the country witnessed the explosive epidemic pattern of the two most recent (re)emergent arboviral diseases, chikungunya and Zika^19^. Since dengue has become a serious burden in public health in Venezuela, year-to-year variation in the size of epidemics, are of particular concern. Therefore, two questions deserve attention: (i) Is there evidence for particular inter-annual (long-term) cycles in the temporal dynamics of dengue? (ii) If so, is the long-term pattern of dengue associated with climate variability? Understanding the inter-annual variability in the population dynamics of DENV can provide useful insights for disease programs and allow the development of more effective surveillance and early warning systems to predict disease risk in response to changes in climate.

This study attempts, first, to search for confirmation of inter-annual cycles of dengue incidence in a time-series of disease data (2001–2016) from two highly endemic regions of northern Venezuela. Then, following the climate hypothesis, to address whether the local and regional climate variability may account for the observed inter-annual variation patterns of dengue incidence in this tropical epidemiological setting.

## Materials and Methods

### Area of Study

**Figure 1:**
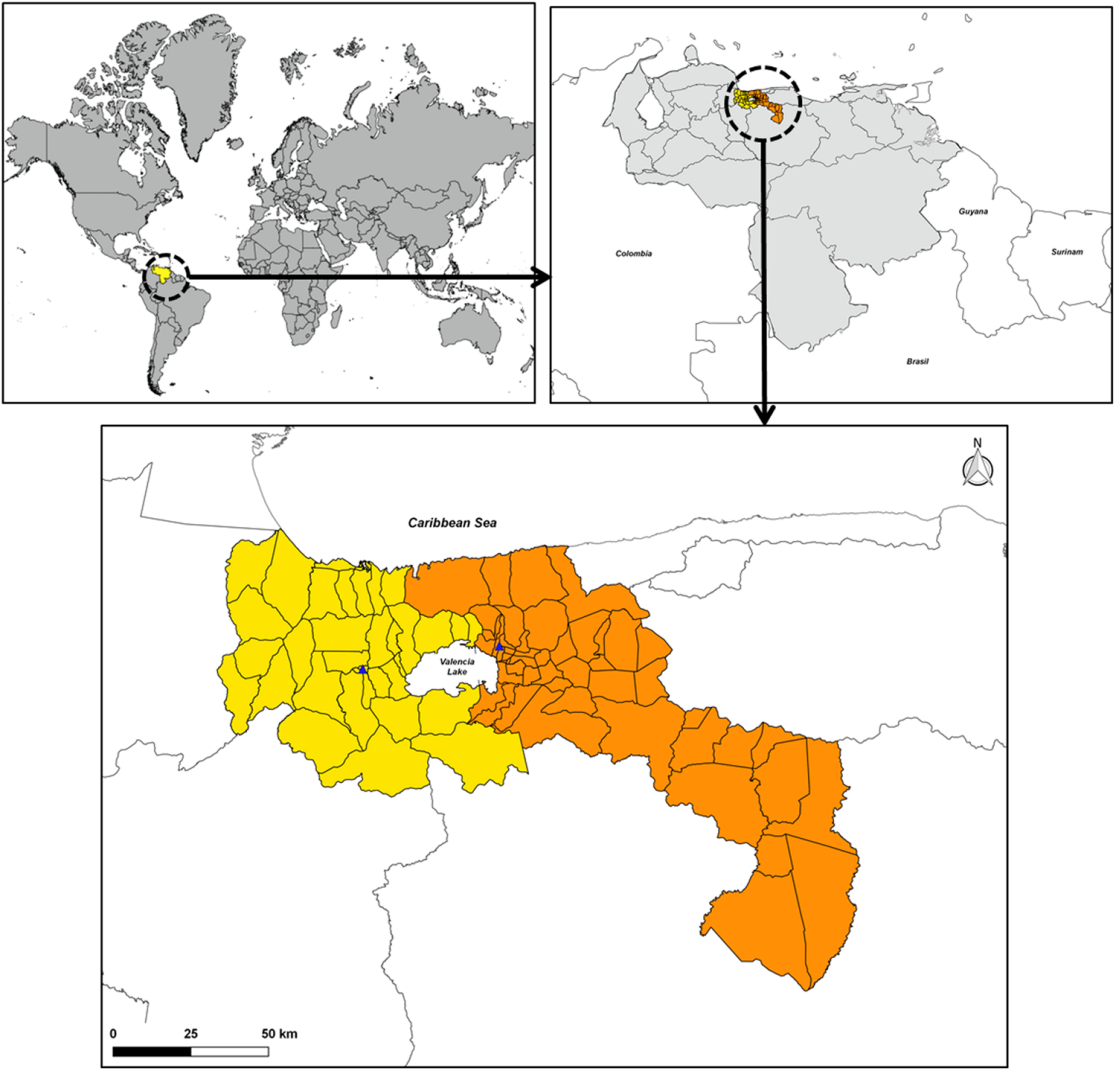
Study area. Map of Venezuela and the northern regions under study: Carabobo (Yellow) and Aragua (Orange) states. Capital city (). Original figure made by Maria Vincenti-Gonzalez with QGIS software (version 2.18, http://www.qgis.org/).

Aragua and Carabobo states are located next to each other in the north-central part of Venezuela (Fig. 1). Both regions are amongst those exhibiting the highest dengue incidences in the country^20^. Regarding the overall local climate, these regions show a mean annual temperature ranging from 23 °C to 26 °C and an accumulated annual precipitation of 700–1199 mm. Since Venezuela is under the influence of the atmospheric phenomena called Intertropical Convergence Zone which drastically affects annual rainfall pattern, the country has a characteristic dry (November-April) and rainy (May-October) season^21^. Both states have an estimated population of 1,805.185 (Aragua) and 2,442.823 (Carabobo) inhabitants^22^. Their capitals, Maracay and Valencia city, comprise the main metropolitan areas of each state, with an urban landscape that varies greatly, ranging from pre-planned urban zones with full access to public services to poor settlements/slum areas where tap water and electricity are unreliable or non-existing. The third most important harbour of Venezuela, “Puerto Cabello”, where major import/export activities occur, is located within the north coastal district of Carabobo state.

### Dengue and climatic data

Countrywide and regional annual dengue cases and incidence were plotted in order to picture the disease long temporal pattern across 26 years (1991–2016) of available records^20^. Additionally, 16 years of available monthly epidemiological data (2001–2016) were analysed to explore the variation and disease periodicity at a finer temporal scale. To calculate monthly and yearly dengue incidence per 100,000 inhabitants, available and projected yearly population data from each state under study was used^22^.

Monthly measures of weather variables were obtained from 2 local meteorological stations: i) Arturo Michelena International Airport (10°08′59′′ N, 67°55′42′′ W) for Carabobo state; and ii) Maracay Base Aérea Sucre (10°15′00′′ N, 67°38′58′′ W) for Aragua state. Local climate data included monthly records of the mean, minimum, and maximum temperature (°C), and the monthly total precipitation (mm).

The El Niño Southern Oscillation (ENSO) is the strongest inter-annual climate cycle on Earth^23^. It is an atmosphere-ocean coupled system that produces quasi-periodic short-term climate and sea surface temperature (SST) changes over the Pacific region with an impact on weather patterns in several countries of the Americas, Africa, and Asia^24^. SSTs are used as an index of ENSO. This climate system oscillation occurs approximately every 3–7 years^23,24^, fluctuating between two extremes known as El Niño corresponding to the warm phase (positive SST anomaly), and La Niña, the cooling phase (negative SST anomaly). El Niño, in fact, refers to the unusual warming of SST. ENSO can be characterized using the Niño 3.4 index, the SST anomaly in the Niño 3.4 region of the equatorial Pacific. El Niño event in turn can be classified according to the SST anomaly index into three categories: weak (0.5 to 0.9 SST anomaly), moderate (1.0 to 1.4 SST anomaly) or strong (>1.5 SST anomaly)^25^. The onset of El Niño events occurs during spring in the Northern Hemisphere, encompassing two calendar years^26^. The events are generally characterized by positive anomalies of SSTs that increase during the Northern Hemisphere’s spring, summer and fall of the first year (Year 0), with the maximum SST anomalies occurring during the winter months (December-January-February) of the following year (Year + 1) and SST anomalies decreasing during the spring and summer of the year + 1. El Niño is the main cause of the inter-annual variability of local climate in the central and eastern tropical Pacific Ocean including the northern coast of South America^26^ and Venezuela^21^. In Venezuela, El Niño is related with negative anomalies of precipitation, soil moisture and river flows, along with positive (warmer) air temperature anomalies^27^ while La Niña has the opposite effect, with cooler temperatures and positive anomalies of precipitation. Here, we used the monthly SST of the eastern and central tropical Pacific as an index of ENSO-region 3.4 (Niño 3.4 index). The SST time-series were obtained from the Climate Prediction Center of the National Oceanic and Atmospheric Administration^28^.

### Time series analyses of dengue incidence and climatic variables

Because disease and climatic time-series, as well as their associations, can be strongly non-stationary (varying in time), a specialized time series analysis method known as wavelet analyses (WA) was applied to detect the periodic cycles and dominant components (i.e. the most frequently repeated signal) of the time series and how they change over time^29,30^. In addition, wavelet coherency (WC) methodology was used to compare the frequency components of dengue and climate time-series in order to quantify the statistical (linear) association between variables in a time span^29^. WC provides local information on when two non-stationary signals are linearly correlated and at what particular frequency.

Additionally, the linear relationship of monthly dengue incidence with monthly observations of climatic variables were also explored through cross-correlation functions (CCF). Finally, we calculated the standardized anomalies of dengue incidence, precipitation and temperatures of our study period by subtracting from each seasonal observation (e.g., **D**ec**J**an**F**eb, **M**ar**A**p**M**ay, **J**un**J**ul**A**ug, **S**ep**O**ct**N**ov) the long-term (16years) mean value of each particular season and dividing this by the long-term seasonal standard deviation.

Data were normalized previous to the analyses. Time series analyses were performed using R (R Foundation for Statistical Computing. Vienna, Austria. Version 3.3.2; 2016), while WA and WC analyses were performed using Matlab 2017a (The MathWorks, Inc., Natick, Massachusetts, United States) and the toolboxes developed by Cazelles *et al*.^15^.

### Data availability

Both dengue and climatic data are publicly available online (Figshare 10.6084/m9.figshare.5799378) as excel files.

## Results

### Temporal patterns of dengue incidence

**Figure 2:**
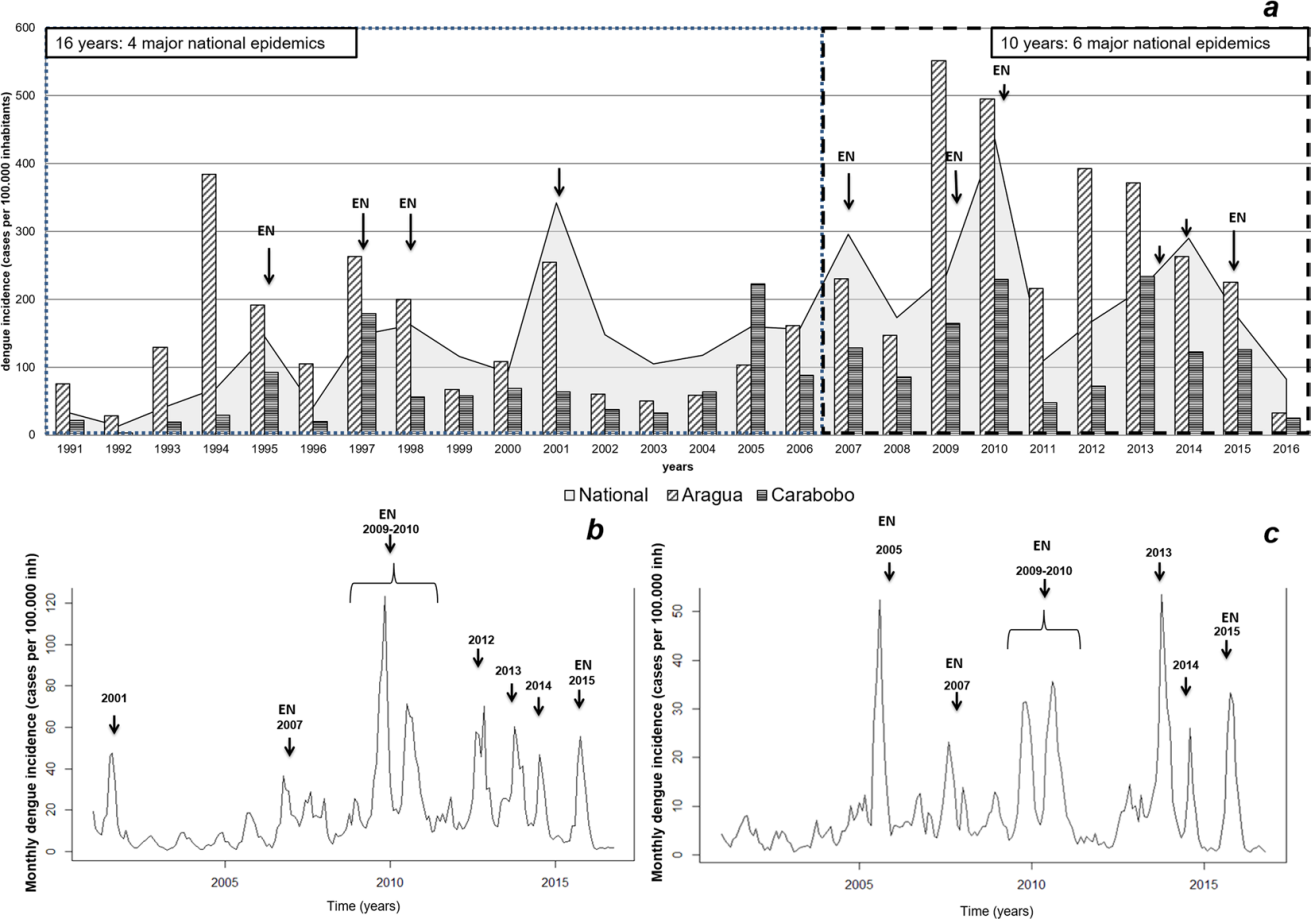
National and regional dengue incidence. (**a**) Annual national dengue incidence. Monthly dengue incidence of (**b**) Aragua and (**c**) Carabobo states. Arrows indicate epidemic years. Dengue epidemic years coinciding with an El Niño event are identified with an ‘EN’ label.

Overall, dengue incidence in Venezuela has exhibited a steady average increase of approximately 9.5% annually between 1991 and 2016 with an average of 39.5 cases x 100,000 inhabitants in the early 1990’s to a 10-fold higher mean incidence of 368 cases × 100,000 inhabitants in the last 6 years (2010–2016). An average national incidence of 157 cases × 100,000 inhabitants (range 13–438 cases × 100,000 inhabitants) was observed during these 26 years (Fig. 2a and Supplementary Figure S1a). Moreover, from 2007 onwards, a total of six epidemic years (years 2007, 2009–2010, 2013, 2014, 2015) were recorded nationally with an intensification in the frequency and magnitude of these outbreaks in comparison with only 4 epidemic years in the previous 16 years (years 1995, 1997–1998, 2001). Overall, most of these epidemic years coincided with an El Niño event (Fig. 2a). Time series of monthly dengue cases (2001–2016) from Aragua and Carabobo regions mirrored the temporal pattern of the whole country. Six major outbreaks encompassing eight epidemic years (2001, 2007, 2009–2010, 2012–2013, 2014 and 2015) were observed in Aragua (Fig. 2b), whereas similar events were registered in Carabobo (Fig. 2c).

**Figure 3:**
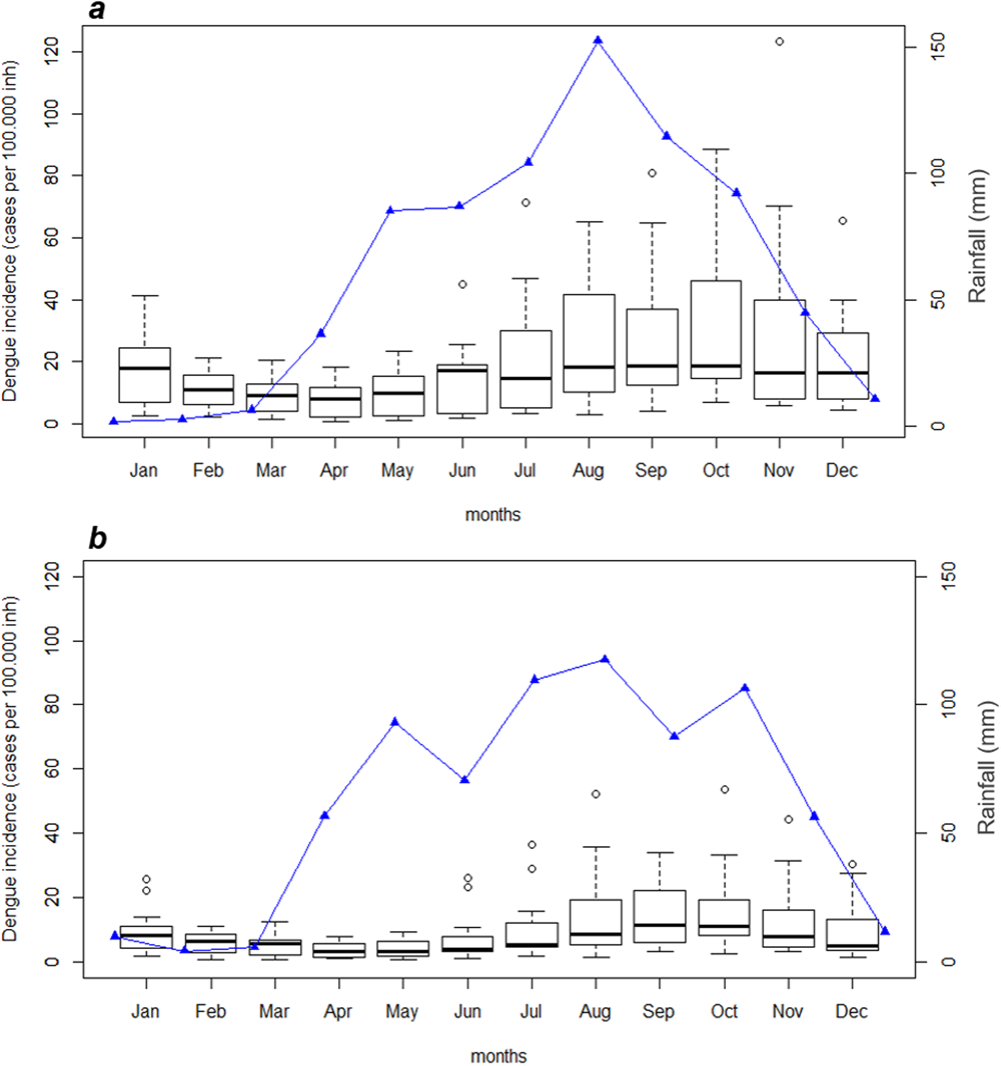
Average monthly dengue incidence (boxplot) and precipitation (blue line) for the period of 2001–2016. (**a**) Aragua and (**b**) Carabobo states.

The average monthly dengue incidence and precipitation in Aragua and Carabobo regions during the period of 2001–2016 are shown in Fig. 3. Dengue shows a clear seasonal pattern coinciding with the rainy season and peaking between August and November. Although Aragua and Carabobo regions are next to each other, the reported average dengue incidence of the past 26 years is 2.25 times as high in Aragua (199 cases × 100,000 inhabitants) as in Carabobo (88 cases × 100,000 inhabitants).

### Periodicity of dengue and climatic variables

**Figure 4:**
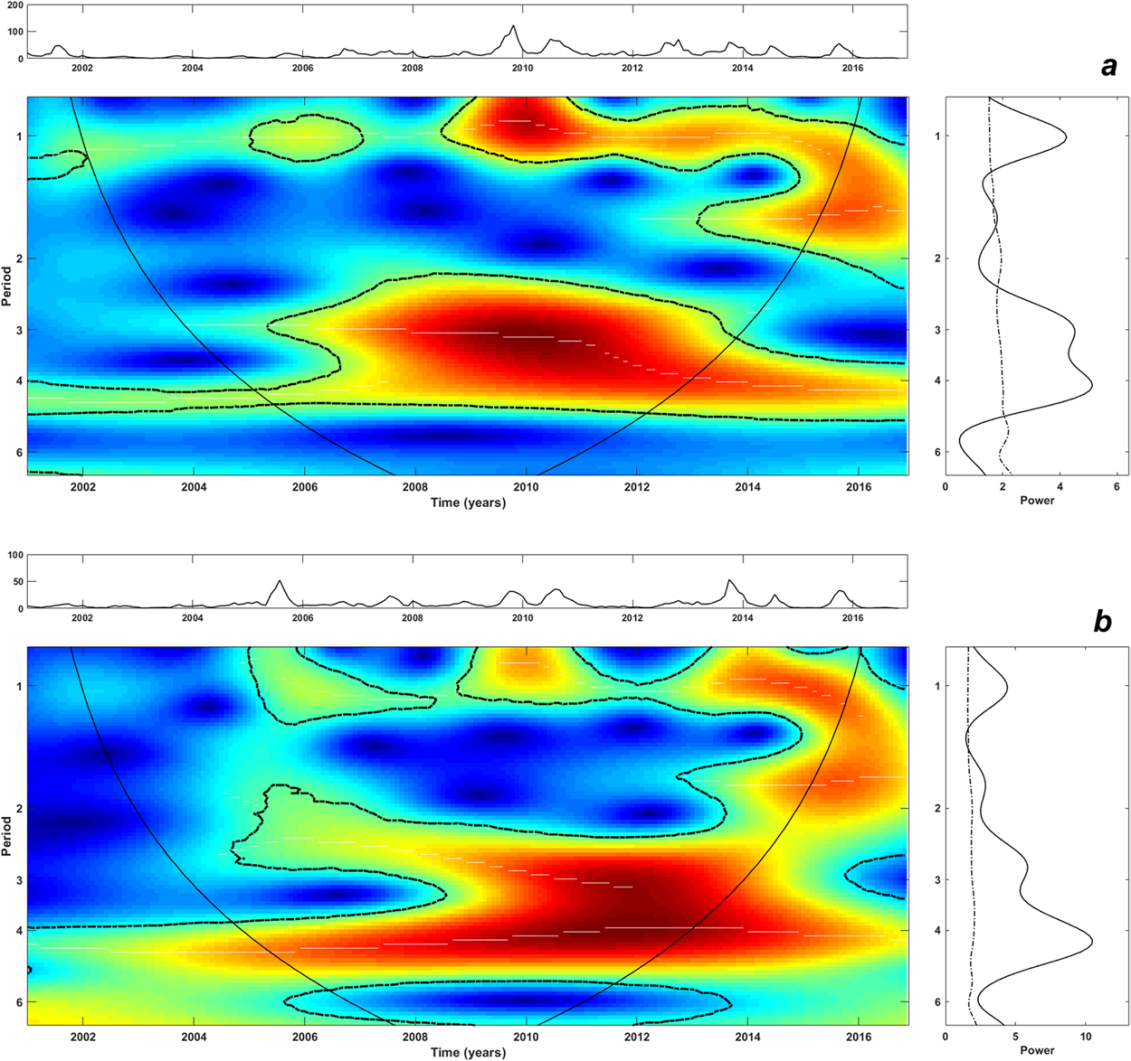
Pattern of inter-annual variability of the monthly time-series of dengue incidence of (**a**) Aragua and (**b**) Carabobo regions. Main central panels: wavelet power spectrums (WPS) of dengue incidence. Right panels: Global spectrum (GS). Top panel over each WPS: Original time-series of dengue incidence data. The *y*-axis of the WPS and GS describe the periods in years (e.g., period 1: annual cycles; period 2,3,4: inter-annual cycles). The *x*-axis of the GS shows the power at a given frequency (continuous line) with its significant threshold value of 5% (dashed line). In the WPS, the color code for power values ranges from dark blue for low values, to dark red for high ones. The areas surrounded by dotted-dashed lines are those including significant results (p < 0.05). The area within the cone of influence (continuous line) in the WPS indicates the region not influenced by edge effects.

Oscillations of dengue incidence at 3–4-years and 1-year scales (cycles) were identified and dominated the disease dynamics in the analysed time-series (Fig. 4). However, these cycles were transient and varied in power. Strong and significant inter-annual cycles peaking around the 3–4-year frequency in Aragua and Carabobo were more pronounced after 2006–2007 (Fig. 4a,b) coinciding with the major dengue outbreaks of that period in the two regions (Fig. 2). The seasonal cycle had less power showing its highest intensity from 2008–2009 onwards (Fig. 4a,b).

**Figure 5:**
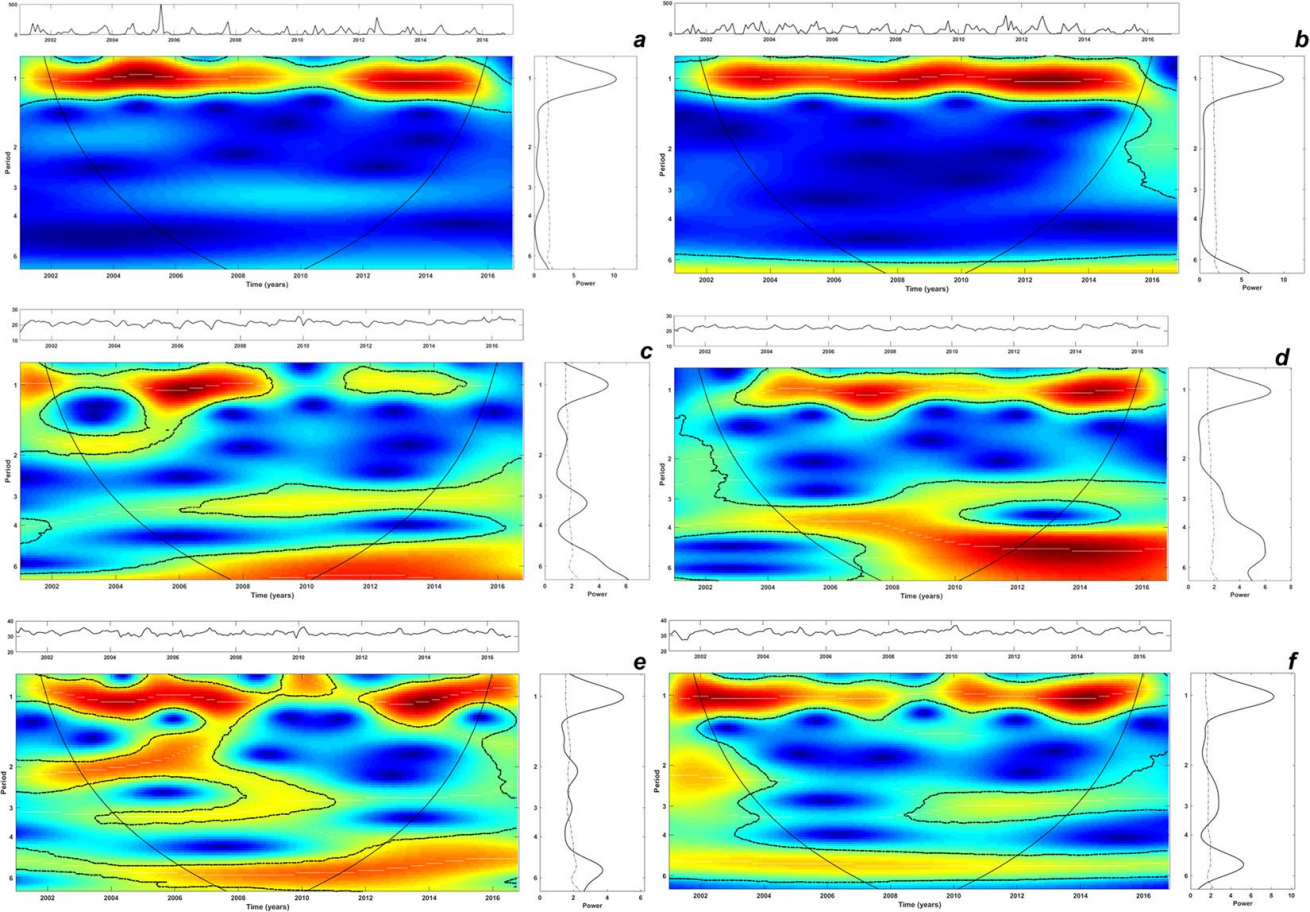
Patterns of interannual variability of the (**a**) monthly rainfall, (**c**) minimum and (**e**) maximum temperatures of Aragua region. Corresponding WPS for (**b**) rainfall, (**d**) minimum and (**f**) maximum temperature of Carabobo region. Main central panels: wavelet power spectrums (WPS) of rainfall and temperatures. Right panels: Global spectrum (GS). Top panel over each WPS: Original time-series of the corresponding variable: rainfall, minimum and maximum temperature. The *y*-axis of the WPS and GS describe the periods in years (e.g., period 1: annual cycles; period 2,3,4: interannual cycles). The *x*-axis of the GS shows the power at a given frequency (continuous line) with its significant threshold value of 5% (dashed line). In the WPS, the color code for power values ranges from dark blue for low values, to dark red for high ones. The areas surrounded by dotted-dashed lines are those including significant results (p < 0.05). The area within the cone of influence (continuous line) in the WPS indicates the region not influenced by edge effects.

**Figure 6:**
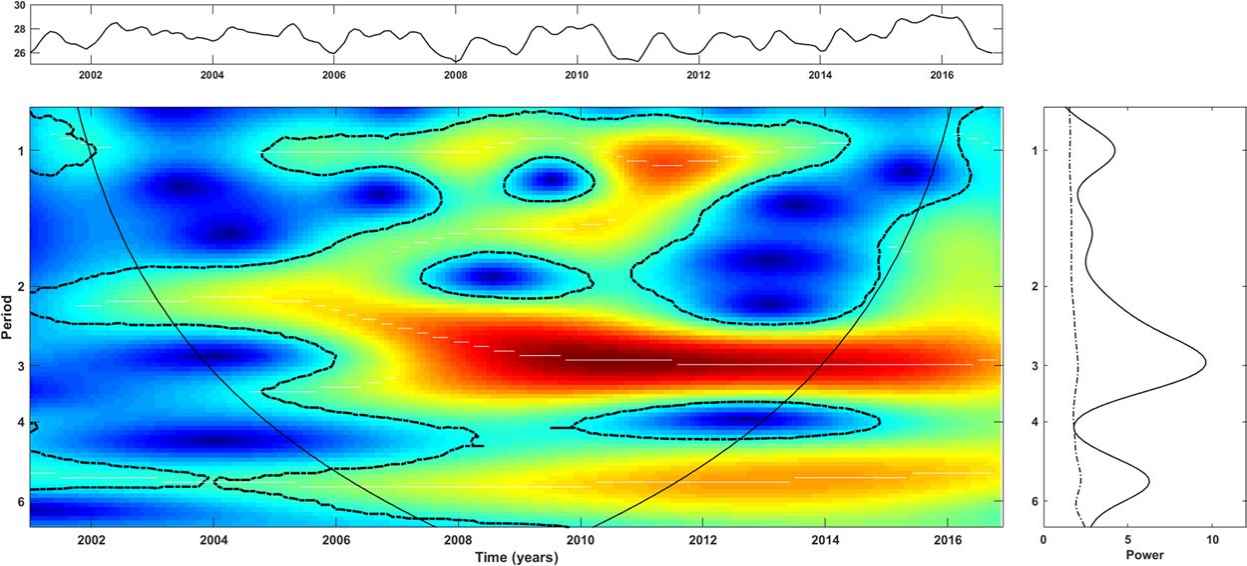
Patterns of interannual variability of the monthly sea surface temperatures (2001–2016). Main central panels: wavelet power spectrums (WPS) of monthly observations of SST. Right panels: Global spectrum (GS). Top panel over each WPS: Original time-series of SST data. The *y*-axis of the WPS and GS describe the periods in years (e.g., period 1: annual cycles; period 2,3,4: inter-annual cycles). The *x*-axis of the GS shows the power at a given frequency (continuous line) with its significant threshold value of 5% (dashed line). In the WPS, the color code for power values ranges from dark blue for low values, to dark red for high ones. The areas surrounded by dotted-dashed lines are those including significant results (p < 0.05). The area within the cone of influence (continuous line) in the WPS indicates the region not influenced by edge effects.

The wavelet power spectrum (WPS) of local rainfall and temperature exhibited strong significant power at a 1-year scale (seasonal) as expected. Moreover, inter-annual cycles at 3–6-years and 6-years were also detected for minimum and maximum temperatures, respectively (Fig. 5). Interestingly, a significant upward trend over time of the minimum and maximum temperatures was observed in both of the regions under study (Supplementary Figure S1b–e). The periodicity of ENSO is depicted across monthly SST records in Fig. 6. This time-series exhibited inter-annual variability at 2–3 and 5-years cycles during the time period of our study.

### Relationship between dengue and climatic variables

**Figure 7:**
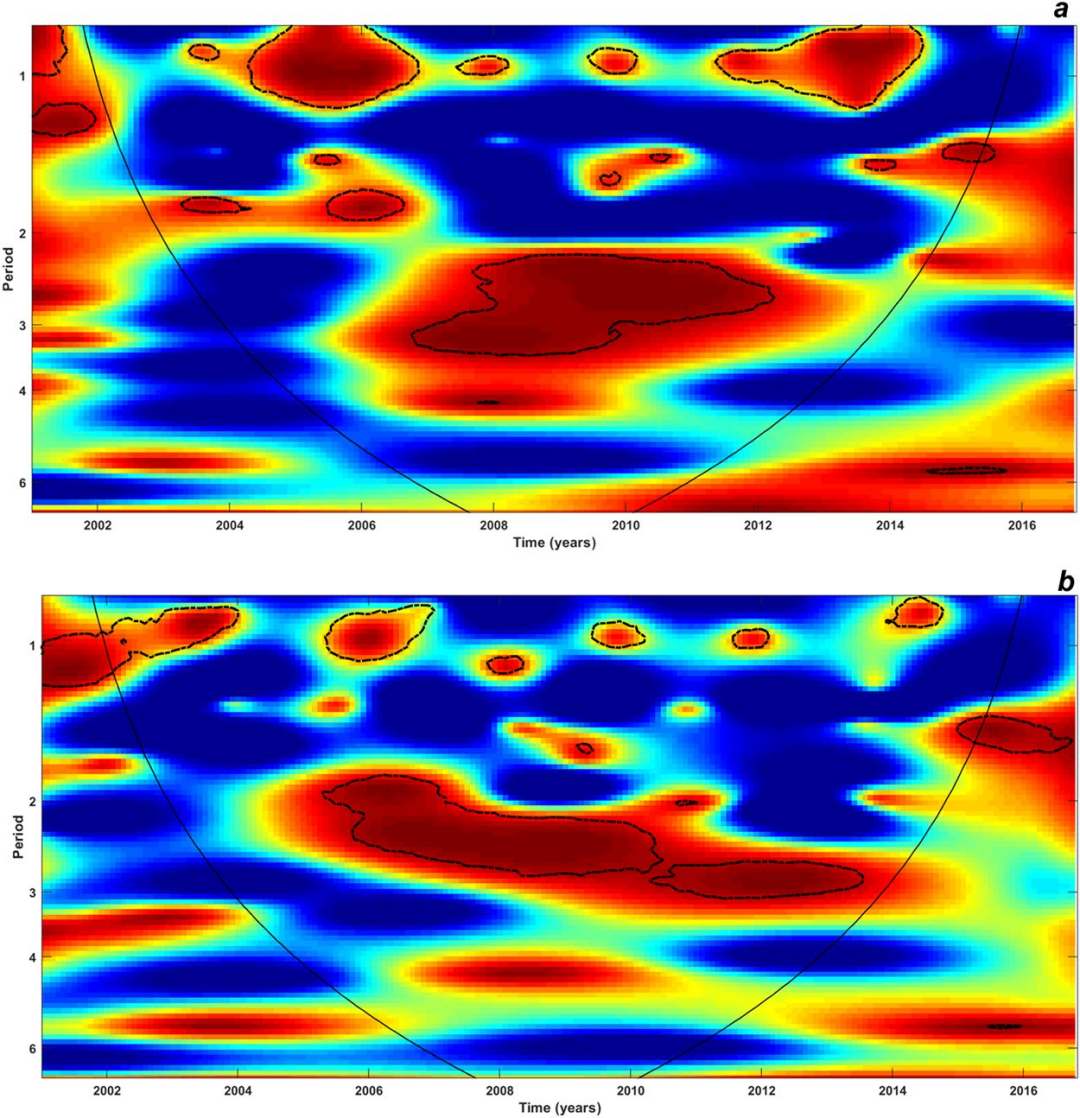
Wavelet coherence spectrum (WCS) of dengue incidence with sea surface temperature (SSTs) time-series for (**a**) Aragua and (**b**) Carabobo. The colors are coded as dark blue, for low coherence and dark red for high coherence between SST and dengue incidence time series. The *y*-axis of the WCS describe the periods in years (e.g., period 1: variables cohered at annual cycles); period 2,3,4: variables cohered at inter-annual cycles). The areas surrounded by dotted-dashed lines are those including significant results (p < 0.05). The cone of influence (continuous line) in the WCS indicates the region not influenced by edge effects.

We evaluated the correspondence of the WPS of dengue and SSTs through WC analysis. As Fig. 7 shows, there was a significant but non-continuous coherence between both variables at 1-year and 2- to 3-year scales across the two studied regions. In Aragua state, dengue incidence mainly cohered with SST from 2007 to 2012 at 2- to 3-year cycles (Fig. 7a). In this region, the 1-year coupling between both signals was stronger than in Carabobo region where instead, the link between dengue and ENSO showed a longer (2005–2013) and significant result at the 2- to 3-year scale (Fig. 7b). There were significant but transient coherences between dengue incidence and rainfall (Supplementary Figure S2a,b), and dengue incidence with minimum (Supplementary Figure S2c,d) and maximum temperatures (Supplementary Figure S2e,f) at the annual (1 year) and 2- to 3-year scales across the two regions. In order to explore the role of inter-annual variations of ENSO on the local climate, WC analysis of these variables were performed tackling the regions under study (Supplementary Figure S3). All climatic variables signals cohered with SST-time series at the seasonal (annual) scale. Furthermore, local climate variables transiently coincided with SSTs signals at the 2- to 6-year scale.

**Figure 8:**
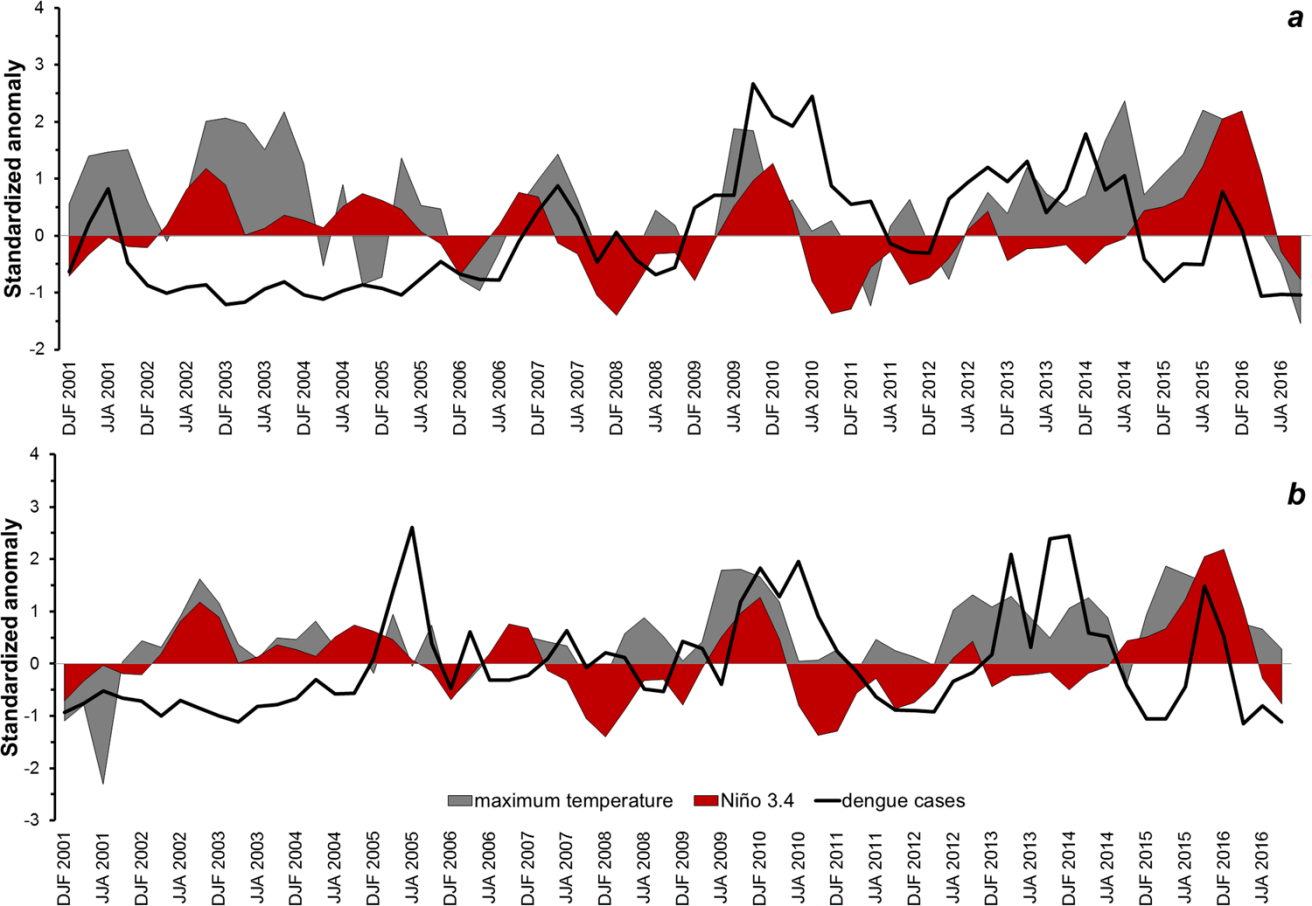
Standardized anomalies of maximum temperature, Niño 3.4 and dengue cases in (**a**) Aragua and (**b**) Carabobo regions.

Figure 8 shows the plots for standardized anomalies of maximum temperature, SST and dengue cases in the regions of interest. Accordingly, five El Niño events took place during our study period: 2002–2003 (moderate), 2004–2005 (weak), 2006–2007 (weak), 2009–2010 (moderate) and 2014–16 (strong, Mega Niño). The 2014–2016 El Niño event is one of the strongest ever recorded. Likewise, five La Niña events occurred during the following periods: 2000–2001 (weak), 2007–2008 (moderate), 2010–2011 (moderate), 2011–2012 (weak), 2016–2017 (weak). We found for both nation-wide and Aragua region that four out of seven disease epidemics coincided with an El Niño episode from 2007 to 2015 (Figs 2a and 8a). This association was found specifically on the dengue epidemic years of 2007 (El Niño 2006–2007), 2009–2010 (El Niño 2009–2010), and 2015 (El Niño 2014–2016). Similarly, for Carabobo region, five out of seven epidemics co-occurred with an El Niño episode. Here, the relationship was detected specifically for the dengue epidemic years of 2005 (local dengue epidemic year, El Niño 2004–2005), 2007 (El Niño 2006–2007), 2009–2010 (El Niño 2009–2010) and 2015 (El Niño 2014–2016) (Fig. 8b). Positive anomalies of maximum temperature corresponded with anomalies of ENSO. We also found that positive anomalies of dengue cases (epidemic years) coincided with maximum temperature anomalies. The previous is noteworthy, since some epidemics that did not coincide with ENSO, concurred with positive anomalies of maximum temperatures. This correspondence between dengue and maximum temperatures occurred in almost all epidemic years. In Aragua region (Fig. 8a) this relationship was present for the epidemic years of 2001, 2007, 2009–2010, 2013, 2014 and 2015; while in Carabobo region (Fig. 8b), this association occurred on the epidemic years of 2005, 2009–2010, 2013, 2014 and 2015–2016.

Cross Correlation Functions (CCF) were calculated to identify relevant associations between dengue incidence and climatic variables at a finer temporal scale (monthly). There was no difference in time lags between both Aragua and Carabobo regions for all CCF. Significant correlations between dengue and SSTs were found at similar lags of 4–5 months for both regions, with highest correlation values at lag 4 (*r* = 0.14) and lag 5 (*r* = 0.25) for Aragua and Carabobo, respectively. Likewise, dengue had a significant and positive association with rainfall taking place two months earlier than dengue in Aragua and Carabobo (*r* = 0.23 and *r* = 0.32, respectively). Finally, high frequencies of dengue incidence were positively correlated with high values of maximum temperatures occurring six months previously for Aragua (*r* = 0.15, p < 0.05) and 6–7 months previously for Carabobo (*r* = 0.36; *p* < 0.05), whereas similar associations were found between dengue incidence and the minimum temperature at lags of 3 (range 3–5) and 5 (range 3–6) months (Aragua: *r* = 0.26 and Carabobo: *r* = 0.29; *p* < 0.05).

## Discussion

We identified long-term (inter-annual) and short-term (seasonal) cycles of dengue incidence in two highly-endemic regions of Venezuela. A strong and significant association was found between dengue inter-annual cycles and ENSO, suggesting that El Niño events have been partly responsible for the periodic outbreaks of dengue in northern Venezuela. We propose that the mechanism of ENSO in driving dengue inter-annual cycles is via warmer local temperatures and lower precipitation. In the studied period, these results were evident in relevant epidemic years, including epidemics of great magnitude, such as the one of 2009–2010, the largest dengue epidemic ever recorded in Venezuela.

Our analysis showed that dengue epidemics at a cyclic frequency of 3–4 years were characteristic of the dynamic of the disease besides its expected 1-year seasonal cycle. Previous studies have documented the occurrence of inter-annual fluctuations in dengue incidence. However, the periodicities reported were different, ranging from 2–3 years^8,15,31,32^ to 2–5 years^9^.

Inter-annual cycles of dengue have been related to several environmental (extrinsic) and immunological (intrinsic) determinants^15,17,18,33^ which affect both vector and virus^7,24^. Here, however, we focused on characterizing the degree of influence of the ENSO regional event and local meteorological conditions on the temporal disease dynamics. We found a strong and significant coherence between the temporal pattern of dengue and that of these variables showing the role that climate plays in driving disease periodicity. Specifically, we identified a relationship between dengue incidence and SSTs at 2- to 3-year cycles across the two studied regions indicating the significant influence of El Niño in shaping the cycle of dengue epidemics in northern Venezuela. A similar association was also detected between dengue and local climate variables such as minimum and maximum temperatures, with a less clear link observed between dengue and precipitation.

At a regional scale, ENSO has been the most commonly studied driver of cyclic climate phenomena in human diseases^34^. In tropical South-America, this event is a periodic climatic oscillation with an average occurrence of 3–7 years, as our analyses showed, and with a strong influence on the inter-annual variability of local climate across different geographical areas^25,34,35^. In Venezuela, temperatures tend to increase by an average of 0.5 °C during El Niño, and severe droughts and negative anomalies of soil moisture, river discharges and rainfall are registered during this climatic episode, the warm phase of the ENSO event. Consequently, warmer and dryer years occur compared with no-Niño years^35^. Inversely, La Niña event shows the opposite effect^25,26^. In agreement with similar observations made in Colombia^36^, our results showed that four out of seven dengue epidemics at national level and in the two studied regions coincided with El Niño events, including a long and extreme dengue outbreak from 2009–2010 which coincided with Niño 0 and Niño +1 years.

Although several studies have tried to disentangle the effects of ENSO on dengue and other vector-borne diseases, this topic is still an area of active investigation together with the impact of climate change on infectious diseases^15,33,36–40^. A plausible mechanism to explain the influence of ENSO on dengue is related to the fluctuations of local climate conditions that are exhibited during an El Niño event^15,33,36^. By analysing this pathway, we found that temperatures and rainfall periodicities corresponded to the interval of observed periodicity in ENSO (2–6 year periods). Likewise dengue inter-annual cycles corresponded roughly to those of temperature (especially maximum temperature) and rainfall within each region. Likewise, dengue epidemics coincided with positive anomalies of maximum temperatures, and negative anomalies of precipitation. Additionally, positive anomalies of maximum temperatures overlapped with an El Niño event, except for those anomalies that were registered between 2011 and early 2014. Our data is in agreement with that of Gagnon *et al*.^36^ who reported that most of the dengue epidemic peaks happening in Colombia between 1981–1988 were associated with El Niño episodes and warmer temperatures. Interestingly, the local temperature records analysed here showed a significant upward trend. This phenomenon could be linked to an urban heat island effect (UHI), which generally occurs in urban settlements^41^ and could affect the transmission of mosquito-borne diseases as it has been previously reported^42^. Further studies are needed to explore and unravel the relevance of this UHI effect on the increment of dengue in Venezuela during the last years.

How the El Niño event and related temperature and rainfall patterns affect disease transmission is a matter of conjecture. It is known that the relationship between local climate variables (e.g. precipitation and temperature) and dengue transmission can be complex, affecting both the dengue vector and the virus^11^. Cazelles *et al*.^15^ suggested that the relationship between climate and dengue might be clearer with temperature than with precipitation. Higher temperatures affect the growth rate of *Aedes* mosquitoes, accelerate the rate of viral replication within the vector and decrease the length of the reproductive cycle, resulting in more infected female mosquitoes over a shorter period of time^11^ with the risk of major inter-annual dengue outbreaks. Particularly, it has been documented that higher temperatures (i.e. >32 °C) decrease the length of *Ae. aegypti* oviposition cycles and egg laying; as a result, the female gonotrophic cycle is shorter whereas blood-feeding frequency is greater^43–45^. Other studies have shown that increased temperatures have the effect of diminishing the time of the extrinsic incubation period (EIP) of DENV in *Ae. aegypti*^46–48^. As an example, Watts *et al*.^46^ showed in experimental infections of *Ae. aegypti* with DENV-2 that the EIP shortened from 12 days to 7 days when the temperature increased from 30 °C to 32–34 °C.

The complexity of the relationship between dengue dynamics and a local climatic factor such as rainfall is exemplified in our study by the positive correlations of dengue with rains in the seasonal period and the negative correlations in the 2–3-y periodic cycle. Differently, precipitation has a crucial influence on mosquito development, because it provides a suitable habitat for the stages of the mosquito life cycle that are water-dependent^11,40,49^. Here, we found a significant coherence between dengue and precipitation at 1-year periods confirming that precipitation is an important driver of dengue occurrence at a seasonal scale as it has been extensively reported^31,50,51^. Indeed, the appearance/increase of dengue cases commonly coincides with both an intensification of rain as observed in our study area and with vector abundance as reported elsewhere^31,52–54^. In Venezuela, the occurrence of household-related potential breeding sites (used car tires, litter outdoors) are a risk factor for dengue transmission during the rainy season. Moreover, the lack of reliable piped water supply has prompted an ever more common behaviour of water storage both indoors and outdoors^52,55,56^ especially during dry conditions^57,58^. As a consequence, areas with public service deficiencies or during dry years (i.e. derived from El Niño events or during warming trends) are likely to exhibit a more perennial dengue transmission.

Among the various approaches developed to study non-stationary data, WA is one of the most efficient tools to detect periodicity in epidemiological time series^29^. This technique is more powerful when applied to longer time-series than the one used in our study. Although our findings should be interpreted with some caution in the light of the previous statement, we are confident of the robustness of our overall results. As previously stated, other factors need to be taken into account when explaining inter-annual cycles of dengue, such as population immunity and the (re)introduction of new DENV serotypes^17^. Epidemic outbreaks of dengue fever were first recorded in Venezuela in 1964 and were partly attributed to the (re-)introduction of previously non-circulating DENV serotypes/strains coinciding with increased spread and densities of *Aedes aegypti*^59,60^. However, from 2000 onwards, the 4 DENV serotypes co-circulate in Venezuela^61^ and although the epidemic peak registered in 2001 can be related to the introduction of serotype 3^62^, this peak also coincided with La Niña event. Moreover, after 2000, at least 3 major epidemics occurred with the co-circulation of all DENV serotypes in the same period. Future research should aim to investigate how climate forcing interacts with the effects of cross-immunity and cross-enhancement between serotypes to determine the population dynamics of this arboviral disease in Venezuela. Finally, the epidemics of chikungunya (2014) and Zika (2015–2016) viruses were important epidemiological events that affected a large number of people in Venezuela. During 2014, concomitant chikungunya and dengue epidemics were recorded which may have resulted in a certain degree of misdiagnosis between the two diseases during the first two weeks of the chikungunya epidemic when health personnel were not yet acquainted with this new disease. However, the remarkable clinical picture of chikungunya was quickly recognized and properly diagnosed in the great majority of cases. Regarding the possible epidemic effect of the introduction of Zika virus in 2015, the data showed a steady increase of dengue cases since August 2015, while the first confirmed autochthonous case of Zika was reported by the end of November. Zika epidemic reached its peak in January-February 2016 lingering on during the rest of this year. Therefore, we discard any potential effect of Zika virus epidemic on the dengue time series analysed.

## Conclusions

We present evidence suggesting that ENSO and its related local climatic changes (above-normal temperatures and below-normal rainfall) have been important drivers of the biennial and triennial cycles of dengue in the northern part of Venezuela during the last 16-years. The significant upward trend observed in dengue incidence in Venezuela raises the question of its potential relationship with the concomitant increase in local temperatures in the main urban settings. Thus, future research should investigate how regional and local climate interact with viral transmission and with behavioural and other socio-economic factors to determine the population dynamics of dengue in Venezuela. Finally, our findings provide significant evidence of the relevant effect of climate on dengue dynamics and suggest that the local and regional climatic factors here studied should be included in an early-warning system for dengue and other *Ae. aegypti*-borne viral surveillance and control in Venezuela.

## Electronic supplementary material

Supplementary Information
