# Supplementary material for: ENSO-driven climate variability promotes periodic major outbreaks of dengue in Venezuela
Source: Sci Rep. 2018 Apr 10;8:5727. doi: 10.1038/s41598-018-24003-z (PMC5893565; doi:10.1038/s41598-018-24003-z)
Supplement: Supplementary file 1 — Supplementary Information [file 41598_2018_24003_MOESM1_ESM.pdf]

Supplementary Information

ENSO-driven climate variability promotes periodic major outbreaks of dengue in Venezuela

Vincenti-Gonzalez MF[1]; A Tami[1,3*+]; EF Lizarazo[1]; ME Grillet[2*+]

[1] Department of Medical Microbiology, University of Groningen, University Medical Center Groningen, Groningen, The Netherlands.

[2] Laboratorio de Biología de Vectores y Parásitos, Instituto de Zoología y Ecología Tropical, Facultad de Ciencias, Universidad Central de Venezuela, Caracas, Venezuela.

[3]Departamento de Parasitología, Facultad de Ciencias de la Salud, Universidad de Carabobo, Valencia, Venezuela.

*****Corresponding authors: maria.grillet@ciens.ucv.ve (MEG)**;** a.tami@umcg.nl (AT)

+These authors contributed equally to this work

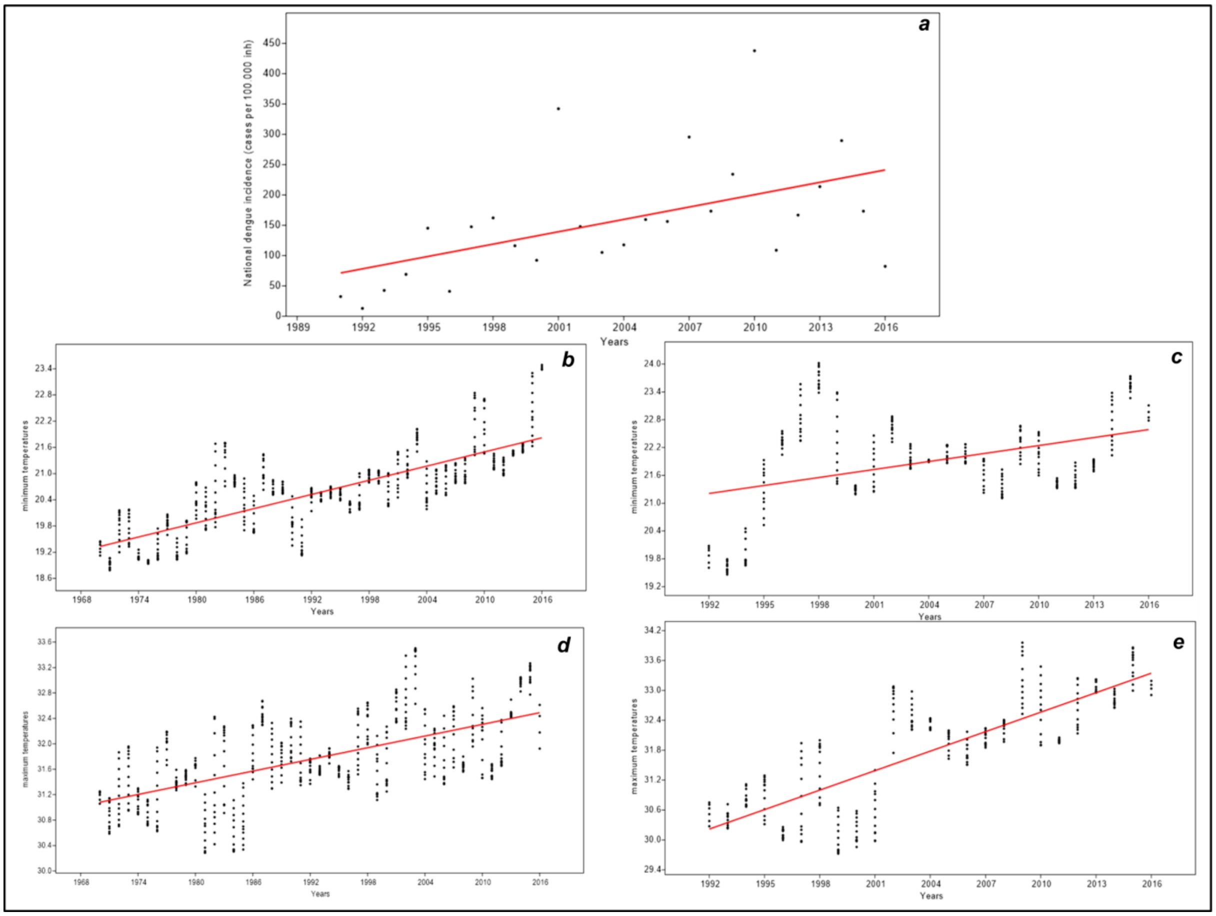

Supplementary Figure S1. National dengue incidence and regional maximum temperature trends. (a) National dengue incidence from 1991 to 2016 (Trend: r^2= 0.27, t = 2.99, p < 0.05, N=26). Minimum temperatures from (b) Aragua (Trend: r^2 = 0.61, t = 29.37, p < 0.05, N=550) and (c) Carabobo (Trend: r^2= 0.15, t = 7.29, p < 0.05, N=286). Maximum temperatures from (d) Aragua (Trend: r^2 =0.36, t =17.93, p < 0.05, N = 550) and (e) Carabobo (Trend: r^2 = 0.65, t = 23.23, p < 0.05, N=286).

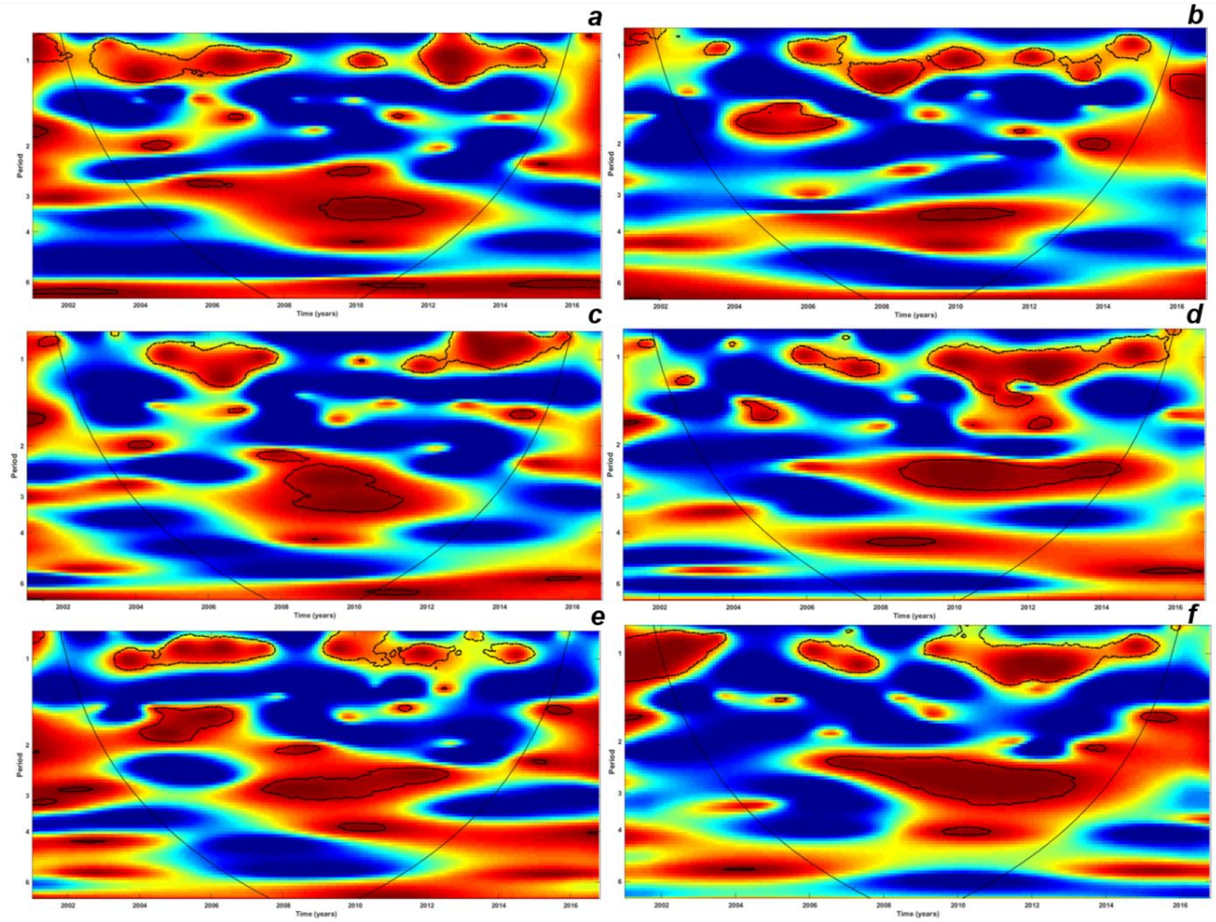

Supplementary **Figure S2. Wavelet coherence spectrum (WCS) of dengue incidence with (a) rainfall, (c) minimum and (e) maximum temperatures of Aragua region. Corresponding WCS for (b) rainfall, (d) minimum and (f) maximum temperature of Carabobo region.** The colors are coded as dark blue, for low coherence and dark red for high coherence between SST and dengue incidence time series. The *y*-axis of the WCS describe the periods in years (e.g., period 1: variables cohered at annual cycles); period 2,3,4: variables cohered at inter-annual cycles). The areas surrounded by dotted-dashed lines are those including significant results ($p<0.05$). The cone of influence (continuous line) in the WCS indicates the region not influenced by edge effects.

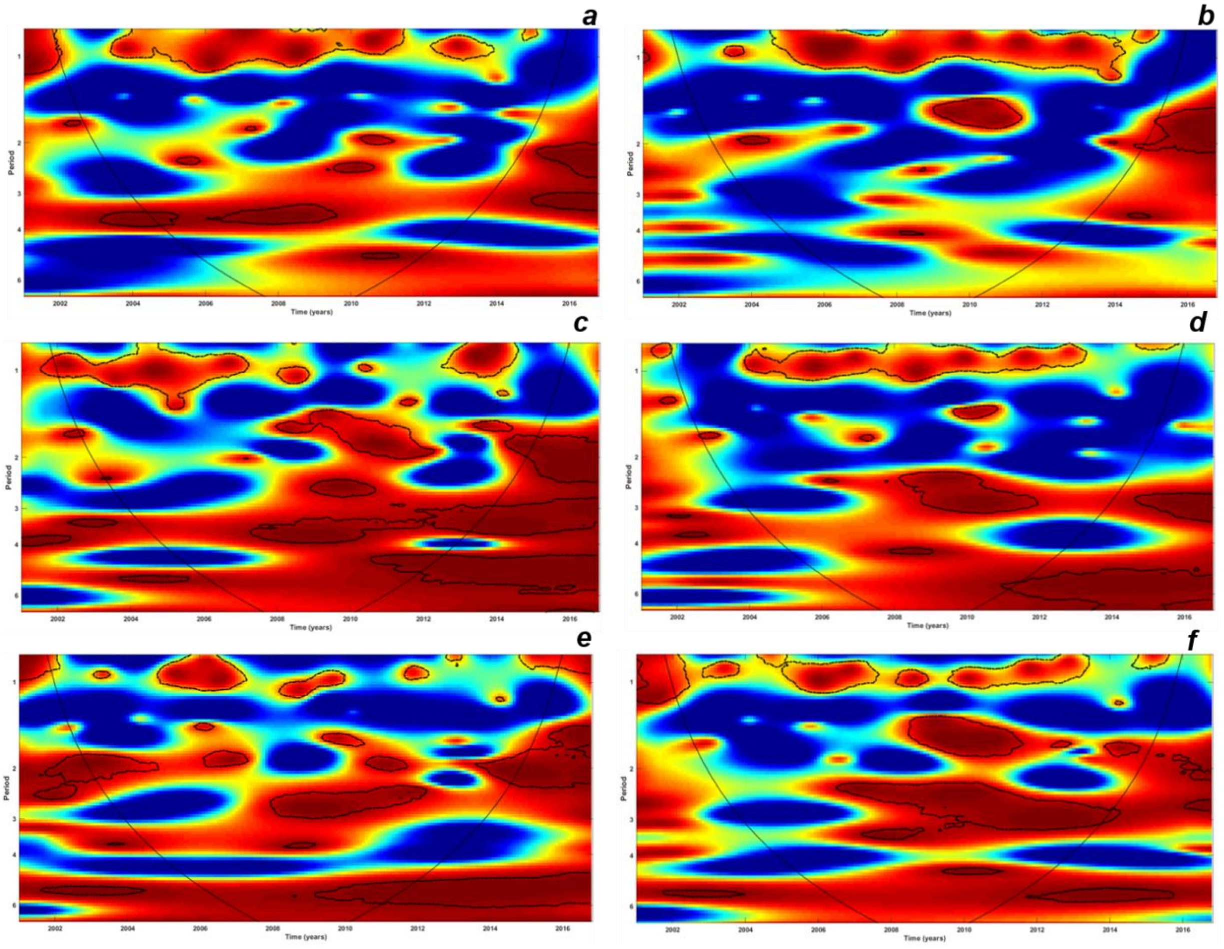

Supplementary Figure S3. Wavelet coherence spectrum (WCS) of SSTs with (a) rainfall, (c) minimum and (e) maximum temperatures of Aragua region. Corresponding WCS for (b) rainfall, (d) minimum and (f) maximum temperature of Carabobo region. The colors are coded as dark blue, for low coherence and dark red for high coherence between SST and dengue incidence time series. The *y*-axis of the WCS describe the periods in years (e.g., period 1: variables cohered at annual cycles); period 2,3,4: variables cohered at inter-annual cycles). The areas surrounded by dotted-dashed lines are those including significant results ($p<0.05$). The cone of influence (continuous line) in the WCS indicates the region not influenced by edge effects.
